# Social support, oral health knowledge, attitudes, practice, self-efficacy and oral health-related quality of life in Chinese college students

**DOI:** 10.1038/s41598-023-39658-6

**Published:** 2023-07-29

**Authors:** Ying Wang, Jie Zhu, Zeling Xu, Xinyi Dai, Keda Chen, Ying Wang

**Affiliations:** 1 Stomatology Hospital, School of Stomatology, Zhejiang University School of Medicine, Zhejiang Provincial Clinical Research Center for Oral Diseases, Key Laboratory of Oral Biomedical Research of Zhejiang Province, Cancer Center of Zhejiang University, Hangzhou, 310000 China; 2 Shulan International Medical College, Zhejiang Shuren University, Hangzhou, 310015 China

**Keywords:** Health care, Health occupations

## Abstract

Oral health is crucial for health-related quality of life. However, the research on the factors affecting oral health status is not comprehensive enough. This investigation aimed to evaluate the multifaceted determinants of college students’ oral health status and explore the impact of social support, oral health literacy, attitudes, behaviors, and self-efficacy on OHRQoL. By surveying 822 students from a university. Baseline data included sociodemographics (gender, age), social support (MSPSS scale), oral health self-efficacy (SESS scale), oral health knowledge, attitudes, and practices (KAP questionnaire), and OHRQoL (OHIP-14 scale). Based on social cognitive theory, partial least squares structural equation modeling (PLS-SEM) and fuzzy set qualitative comparative analysis (fsQCA) were used to examine the relationship between the study variables. PLS-SEM results showed that knowledge, attitude, and practice predicted OHRQoL through self-efficacy. FsQCA results showed that the combination of different variables was sufficient to explain OHRQoL. The conclusion was that self-efficacy plays an important role and the combination of high-level knowledge, positive attitudes, and strong self-efficacy was important in improving OHRQoL. The results of this study provided a reference for the oral health strategy planning of college students in China.

## Introduction

Oral diseases not only have adverse effects on physical, social, and mental health but also limit individual development^1^. A large proportion of the population will face oral and dental-related problems in daily life^2^. The high rate of dental caries^3^, the high proportion of orthodontic^3^ and restorative care^3^ and the low use of oral-health education in adolescence stage^4^ reflect the fact that college students have very specific oral health needs. Although college students have a positive attitude toward the prevention of dental caries and periodontal disease, their understanding of oral diseases is not comprehensive enough^5^. Bad practice formed during adolescence may lead to poor oral health^6^. In addition, poor diet, eating disorders, and other psychosocial problems also affect the oral health^3^. Oral health-related quality of life (OHRQoL) is a multidimensional paradigm involving the subjective evaluation of an individual's oral health, functional health, emotional health, expectations and satisfaction with care, and self-awareness^7^. These affect the oral health-related quality of life. Therefore, exploring the influencing factors of their oral health status has a positive practical significance to improve the OHRQoL.

The factors that affect the OHRQoL are multiple and complex. Health is defined as physical, psychological and social well-being^8^, that is, a healthy bio-psycho-social model, physical function, emotional factors, and social factors will affect individual health^9^. OHRQoL has been used to assess oral health, emotion, and self-esteem to understand the interaction between social factors, environmental factors, physical function, and oral health^7^. From the perspective of biomedical and psychological aspects^10^, some studies have confirmed that psychosocial factors, oral health knowledge, attitudes, practice, and other factors affect oral health to varying degrees^11–13^. Thus, the role and function of oral health knowledge, attitudes, practices, self-efficacy and social support in the OHRQoL need to be investigated.

The Knowledge, Attitude, and Practice (KAP) theory is a theoretical model that changes human health-related behavior^14^. The model mainly originated from social learning theory and innovation diffusion theory^15^. Studies have shown that oral health knowledge, attitudes, and practice all affect oral health quality^13^. Oral health knowledge has a direct and positive impact on attitudes, and knowledge indirectly affects practice through attitudes^13^. In addition, when individuals hold positive attitudes, they are more motivated to implement practice and obtain better results^16^. In previous multidimensional studies, self-efficacy was considered a predictive factor for oral health status^17,18^. The formation of self-efficacy is a long process^19^, mainly derived from an individual's own performance, imitation by others, persuasive language, and emotions related to behavior^20^. Studies have shown that oral health knowledge can change students' oral health practice and self-efficacy^21^. And higher self-efficacy is related to better oral health practice and gingival health^22^.

Social support can convey the basic facts, knowledge, and information that affect emotions, as well as influence an individual's cognitive responses and emotional, behavioral, and health beliefs^23^. Research shows that support from family and friends has a positive impact on adolescent health-related behavior^20^. Children with a lower socio-economic status and poor oral care practice have lower OHRQoL and good oral health practice have a positive impact on OHRQoL^12^. At the same time, parents’ own oral health practice will also affect the oral health of the next generation, which shows that social support has a certain impact on children’s oral health^13^. Knowledge can play a mediating role between social support and self-efficacy in the study of pediatric osteoporosis^24^. Researchers found that older adults with adequate social support had greater access to resources, changed attitudes, and reduced risk of depression^25^. In addition, there is a relationship between low social support and individual depression^21^. The maintenance or change of an individual's health practice is also influenced by social support^22^. Strengthening individual, family, and social resources, as well as individual autonomous coping with health risks and damage, plays an important role in maintaining health of adolescents^26^. Therefore, social support can change an individual's OHRQoL through self-efficacy and health knowledge, attitudes, practice.

**Figure 1:**
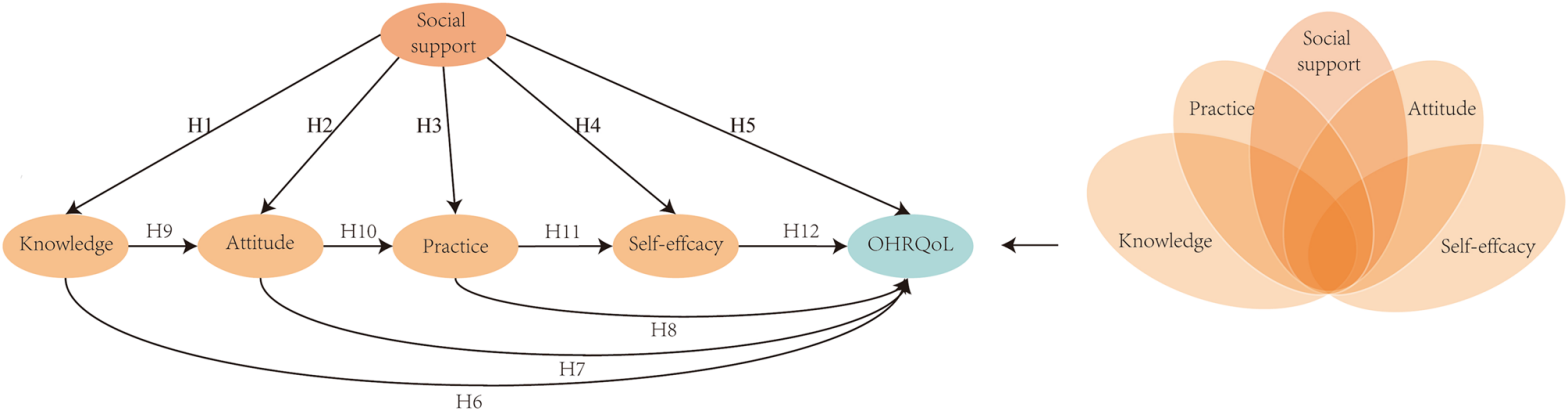
Partial least square structural equation modeling (PLS-SEM) and fuzzy set qualitative comparative analysis (fsQCA) hypothetical model.

Historically, most studies on health-related knowledge, attitudes, and behaviors have focused on evaluating different populations or fields, such as the oral health status of rural children^27^ or the hepatitis prevention status of medical students^28^. Few studies on the relationship between knowledge, attitudes, and habits related to oral health and OHRQoL of university students. In addition, previous studies have indicated that social support^29^, health-related behavior^13^, and self-efficacy^30^ can directly affect OHRQoL, but these studies focused on investing single causal and linear relationships. Facing with the many complex factors that affect OHRQoL, these studies produced contradictory conclusions. For instance, while some studies have shown that mother’s oral health knowledge is associated with children’s oral health^31^, others have demonstrated that while a mother’s knowledge alone may not have a direct impact on children's sound dentition when coupled with a mother’s attitudes, it can produce children's sound dentition an additive effect^32^. The other study shown that some vulnerable social groups in Hong Kong are supported by family members, but the incidence of dental caries is still very high^14^. Therefore, the complex relationship between these factors and OHRQoL has not been fully elucidated. It is necessary to use different methods to study the complex causal relationships between multiple variables that affect oral health. In this study, we use the partial least squares structural equation model (PLS-SEM) to determine the linear (symmetric) causal relationship between influencing factors and OHRQoL, and the fuzzy set qualitative comparative analysis (fsQCA) to determine the nonlinear (asymmetric), heterogeneity, and dynamic interaction between predictive factors and results. As shown in the hypothetical model (Fig. 1), we endorsed the methodologically wiser approaches that combine both analytical techniques for the outcome of interest. Thus, this study was conducted to understand and investigate these complex factors affecting the OHRQoL of college students. The results provide the oral health of college students with a nuanced understanding of the complex causality and trade-off between antecedent factors, helping them devise effective healthy strategies.

## Materials and methods

### Study design and sampling procedures

The study was conducted at a university in Zhejiang Province, China. Baseline data included demographics (gender, age), social support, oral health knowledge, attitudes, practice, self-efficacy and assessed subjects' OHRQoL.

The final sample size was 822 college students, who volunteered to participate in the study. The final sample size was 822 people who volunteered to participate in the study. The inclusion criteria are as follows: (1) there are no reading and comprehension disorders; (2) college students. The exclusion criteria are as follows: (1) incomplete data; (2) mental illness; and (3) reluctance to participate after explaining the study. Because of the nature of the study, the questionnaire is anonymous and protected.

### Conceptual framework

The hypothetical framework of this study is proposed according to previous literature (Fig. 1). At the same time, the following verified tools were selected and adapted for this study (Supplementary table 1). The questionnaire was translated into Chinese and discussed with experts.

### Variables

#### Social support

Using the dimension of the Multidimensional Scale of Perceived Social Support (MSPSS^33^,) as an indicator, social support is a potential variable^34^. The scale was used to measure the social support of family, friends, and partners. The original scale is a scale containing 30 questions, which is simplified to 6 questions in this study. The item was answered on a Likert five-point scale, ranging from “1: Strongly disagree” to “5: Strongly agree”. The total score is obtained by adding up the scores of the items and ranges from 6 to 30. The higher the score, the better the social support the subjects received.

#### Self-efficacy

Using the dimension of the oral health care self-efficacy scale (SESS^35^,) as an index, self-efficacy is the decisive factor affecting the compliance of patients with chronic diseases^36^. The scale was used to measure the degree of self-efficacy in consulting a dentist, cleaning teeth, and eating practice. The original scale is a scale containing 15 questions, which is simplified to 14 questions in this study. A Likert five-point scale was used, ranging from “1: Strongly disagree” to “5: Strongly agree”. The total score is obtained by adding up the scores of the items and ranges from 14 to 70. The higher the score, the higher the oral self-efficacy the subjects received.

#### Oral health knowledge, attitudes, practice

The knowledge, attitudes, and practice of college students were assessed according to the questions used in the oral health knowledge, attitudes, practice (KAP) questionnaire^37^. The original scale is a scale containing 21 questions, which is simplified to 18 questions in this study. The item was answered with the Likert five-point scale, ranging from “1: Strongly disagree” to “5: Strongly agree”. The total score is obtained by adding up the scores of each item. The higher the score, the better the oral knowledge, attitude, and practice the subjects received.

#### Oral health-related quality of life

The scale evaluates the quality of life related to oral health, and it has good psychometric characteristics^38^. The original scale is a scale containing 21 questions. According to the Likert five-point scale, respondents’ responses were “very often = 4”, “often = 3”, “occasionally = 2”, “almost never = 1” or “never = 0”. The total score is obtained by adding up the scores of the items and ranges from 0 to 56. The higher the overall score of OHIP-14, the lower the OHRQoL^13^.

### Data analysis

Confirmatory factor analysis (CFA) and Cronbach's alpha were used to verify the validity and reliability of the questionnaire. To test the hypothesis, this work proposes a conceptual model that includes dimensions that may affect oral-related health. PLS-SEM (V.3.2.9)^39^ was used to analyze the external and internal models to explore the influence of various factors. Each path of the internal model is calculated and evaluated by using a bootstrapping program (the original dataset was 5000 subsamples); and the standard error, T value, and *p*-value are calculated. The fsQCA 3.0^40^ software package was used to perform fuzzy set qualitative comparative analysis. It can be used to test causal and independent variables, and how combining variables leads to the same results as dependent variables.

After checking for missing data, all values between 0 and 1 were then recalibrated. When only two values are considered, 0 (not featured) and 1 (featured) were used. The following thresholds are considered together with continuous variables: 5%, 50%, and 95%^41^, and analysis is performed after the response is changed. FsQCA analysis provides three possible solutions (complex, minimalist, and intermediate solutions). According to the suggestion of the literature^40,42^, we adopted the latter.

### Informed consent

This study was conducted in accordance with the Declaration of Helsinki, and the study protocol was reviewed and approved by the Institutional Review Board of Zhejiang Shuren University (NO: 202201017). Informed consent information was included with each questionnaire and introduced before the surveys. Surveys were only conducted if subjects were fully informed of the content and aim of this research project and agreed to participate.

## Results

### Descriptive statistics

Data collection and analysis were carried out from May to September 2022. Results Questionnaires were sent to 822 students, of which 41 were returned, a response rate of 95.01%. The average age of the respondents was 19.14 years old (SD = 1.01), the range was 17–23 years old, and the proportion of women was 74.20%^43^ (Table 1).

**Table 1:** Participants’ demographic characteristics. (N = 822).

Variable	Category	Frequency	Percentage (%)
Gender	Male	201	25.70
	Female	580	74.20
Age	17–19	676	86.56
	20–23	105	13.44

### Partial least squares structural equation modeling (PLS-SEM)

**Figure 2:**
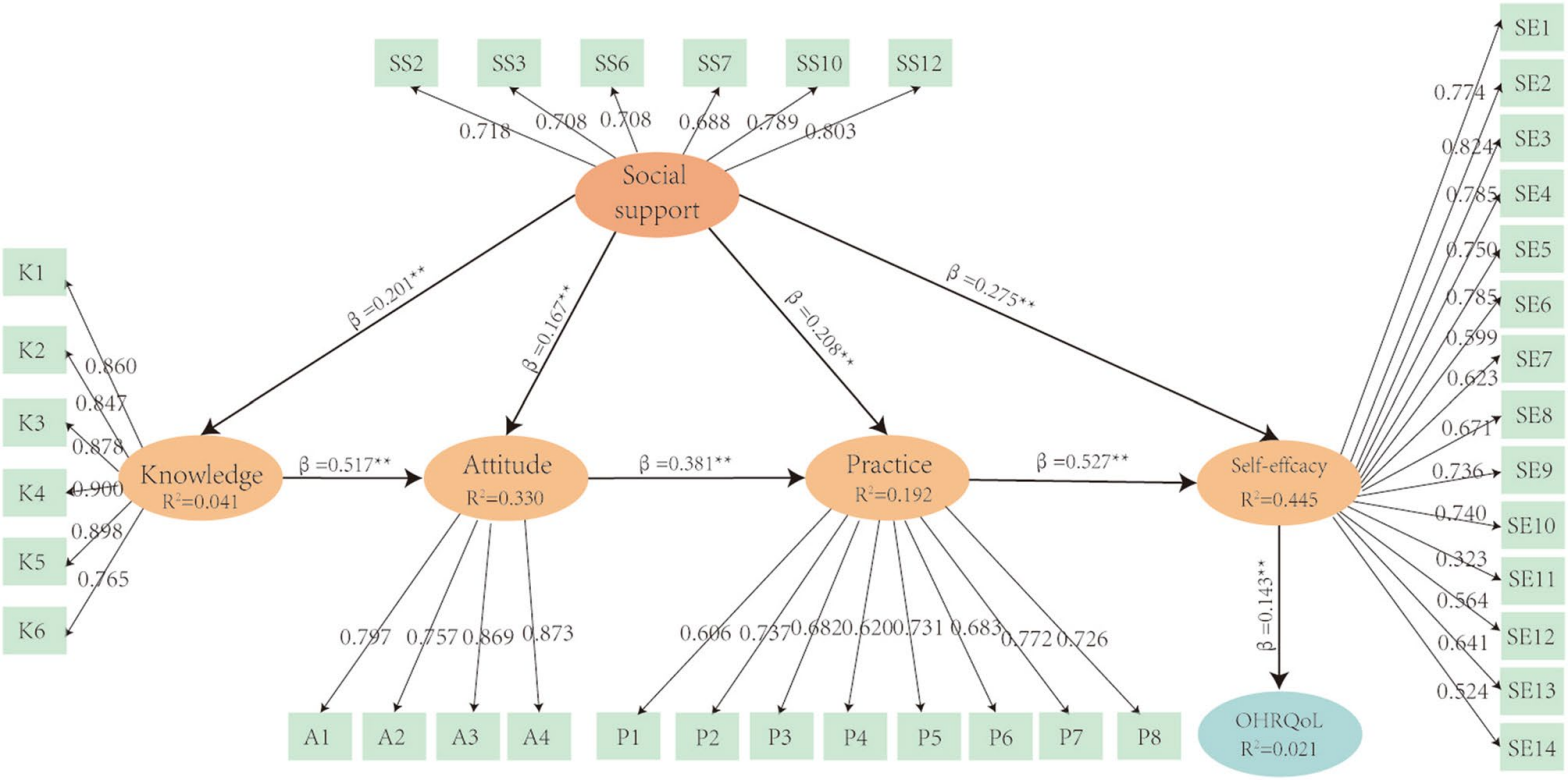
Path model and PLS-SEM. *p < 0.05; **p < 0.01; *SS*: Social support; SE self-effcacy, *K* knowledge, *A* attitude, *P* practice.

Supplementary table 1 illustrates the indicators used by the external model. For all variables, the factor loading range obtained was typically greater than 0.5^44^; and the consistent reliability coefficient of Cronbach's alpha (CA) is always > 0.7^45^, indicating that the internal reliability of its dimension is acceptable. The average variance extraction (AVE) of these dimensions is > 0.5, indicating the effectiveness of convergence^44^. Table 2 determines the importance of each relationship and its impact on the internal model. A value of *p* < 0.05 was considered statistically significant^46^. Specifically, it confirmed the positive effects of various variables (social support, self-efficacy, oral health knowledge, attitudes, practice) on OHRQoL, in which self-efficacy played an intermediary role (Fig. 2). The blindfold procedure show that Q2 is > 0, confirming the predictive relevance of the study model^47^. The SRMR is 0.071. When the SRMR value is less than 0.08, the model fits well^48^.

**Table 2:** PLS-SEM: inner model.

Relationship	Standardized beta	Mean	Standard deviation	*T*-value	*P*	Decision
Social support—> Knowledge	0.201	0.203	0.037	5.457	< 0.001	H1 is supported
Social support—> Attitude	0.167	0.167	0.033	5.041	< 0.001	H2 is supported
Social support—> Practice	0.208	0.209	0.040	5.149	< 0.001	H3 is supported
Social support—> Self-efficacy	0.275	0.276	0.034	8.078	< 0.001	H4 is supported
Knowledge—> Attitude	0.517	0.518	0.038	13.501	< 0.001	H9 is supported
Attitude—> Practice	0.381	0.382	0.035	10.929	< 0.001	H10 is supported
Practice—> Self-efficacy	0.527	0.528	0.031	17.261	< 0.001	H11 is supported
Self- efficacy—> OHRQoL	0.143	0.143	0.031	3.784	< 0.001	H12 is supported

### Fuzzy set qualitative comparative analysis (fsQCA)

#### Necessity analysis

According to the results of the necessity analysis (Table 3), because all the consistency values are below 0.90^49^, no single factor is a necessary condition for oral health.

**Table 3:** Necessity analysis for oral health related quality of life (OHRQoL).

	High OHRQoL		Low OHRQoL	
	Cons	Cov	Cons	Cov
Social support	0.658256	0.67243	0.652649	0.554267
~ Social support	0.563664	0.661239	0.61429	0.5991
Self-efficacy	0.626318	0.709655	0.569836	0.536772
~ Self-efficacy	0.59117	0.623079	0.691771	0.60615
Knowledge	0.686512	0.694854	0.625713	0.526511
~ Knowledge	0.532196	0.631041	0.637362	0.628289
Attitude	0.75653	0.661369	0.715265	0.519843
~ Attitude	0.450758	0.655674	0.534073	0.645851
Practice	0.603692	0.701364	0.575084	0.555452
~ Practice	0.617361	0.636051	0.690811	0.591696

*Cons* consistency, *Cov*, coverage; ~ , absence of condition; Condition needed: consistency ≥ 0.90.

#### Sufficiency analysis

With regard to sufficiency analysis, the combination of conditions affecting OHRQoL was calculated (Table 4). The frequency cutoff value in the truth table is set to 1 and the consistency cutoff value is set to 0.77.

**Table 4:** Combination of conditions affecting oral health-related quality of life (OHRQoL).

	Raw coverage	Unique coverage	Consistency
Causal models from a high level of OHRQoL			
OHRQoL = f(SS*SE*K*A*P)			
C1: SE*K*A	0.492309	0.182291	0.755461
C2: ~ SS*SE*A* ~ P	0.288985	0.0259813	0.827282
C3: SS*SE*K* ~ P	0.295456	0.0231438	0.806144
solution coverage: 0.541434			
solution consistency: 0.753271			
Causal models from a low level of OHRQoL			
~ OHRQoL = f(SS*SE*K*A*P)			
NC: ~ SS* ~ SE* ~ K* ~ A* ~ P	0.388053	0.388053	0.751024
solution coverage: 0.388053			
solution consistency: 0.751024			

SS social support, SE self-efficacy, *K* knowledge, *A* attitude, *P* practice, *OHRQoL* oral health-related quality of life.

The forward solution shows that the combination of three causal conditions can produce better OHRQoL (consistency = 0.75; coverage = 0.54; Table 4). As mentioned earlier, in the sufficiency analysis the original coverage refers to the variance of the interpretation, which means that the number of observations can be explained by a specific combination of conditions. The consistency of the solution indicates the reliability or fitness of the model. In fsQCA, when the overall consistency of the model is ≥ 0.66, the model is valid^50^. Therefore, the solution obtained through fsQCA is effective.

The fsQCA results showed that a combination of better self-efficacy, a positive oral health attitude, and rich oral health knowledge were associated with good OHRQoL (C1 in Table 4, coverage = 0.49; consistency = 0.75). Higher self-efficacy, richer oral health knowledge, greater social support and lower oral health attitude were supported to better OHRQoL (C3 in Table 4, coverage = 0.30; consistency = 0.80). Another combination that was linked to good OHRQoL was having high self-efficacy and a positive attitude towards oral health, even if the individual had poor oral health practices and lower social support (C2 in Table 4, coverage = 0.29; consistency = 0.82).

### Comparsion of PLS-SEM and fsQCA

The study utilized PLS-SEM to determine the positive effects of social support, self-efficacy, oral health knowledge, attitudes, and practices on OHRQoL, with self-efficacy playing an intermediary role. The results also indicated that the average variance extraction (AVE) of these dimensions was > 0.5, demonstrating the effectiveness of convergence. Furthermore, fsQCA identified two combinations associated with good OHRQoL: better self-efficacy, a positive oral health attitude, and rich oral health knowledge; and high self-efficacy and a positive attitude towards oral health, even in individuals with poor oral health practices and lower social support. The fsQCA results reinforce the PLS-SEM results (Table 5).

**Table 5:** Comparison between the results of PLS-SEM and fsQCA.

	Methods	
Hypothesis	Structural equation model (SEM)	Fuzzy set qualitative comparative analysis (fsQCA)
H1	Supported	Conditional supported
H2	Supported	Conditional supported
H3	Supported	Conditional supported
H4	Supported	Conditional supported
H5	Not supported	n.a.^a^
H6	Not supported	n.a.^a^
H7	Not supported	n.a.^a^
H8	Not supported	n.a.^a^
H9	Supported	Conditional supported
H10	Supported	Conditional supported
H11	Supported	Conditional supported
H12	Supported	Conditional supported

^a^Not applicable.

## Discussion

The results of this study show that social support, oral knowledge, attitudes, and practice should use self-efficacy as an intermediary variable to affect college students’ OHRQoL. This is consistent with the results of previous studies^13,51^. In addition, previous studies on the influencing factors of OHRQoL mainly use traditional symmetry and linear relationship analysis, which may ignore the existence of multiple complex causalities among the influencing factors. Therefore, this study not only constructed an SEM model to explore the linear relationship between variables, but also used the fsQCA method to supplement the conclusion of multiple complex causalities.

The PLS-SEM results (Table 2, Fig. 2) show that self-efficacy is the core factor affecting OHRQoL and mediates between other factors and OHRQoL. The fsQCA partially validates the SEM results (Table 5). Although the influencing factors do not necessarily affect OHRQoL, self-efficacy factors are involved in all solutions (C1, C2, and C3 in Table 4). This illustrates the core role of self-efficacy and partially verifies its intermediary role (Table 4), which is similar to previous results^52^. In social cognitive theory, self-efficacy can predict individual behavior specificity, health quality, and well-being; and evaluation of self-efficacy and quality of life is an important part of the core outcome of frequently-occurring diseases^53^.

Similarly, self-efficacy is also an important factor in the self-management of chronic diseases^54–56^, such as hypertension^57^ and diabetes^58^. A study of patients with type 2 diabetes found that patients with high self-efficacy paid more attention to learning relevant knowledge and actively managing the condition^59^. Therefore, we should pay attention to improving individual self-efficacy, especially the level of oral knowledge and cultivating a positive attitude to significantly improve OHRQoL.

At the same time, the complex relationship between self-efficacy and its predictor factors also provides new ideas and insights for improving OHRQoL. Knowledge, attitude, and practice indirectly affect OHRQoL: knowledge indirectly affects practice through attitude, and attitude affects self-efficacy through practice, thus affecting OHRQoL (Table 2). The relationship between knowledge and attitude is similar to a previous study^60^. It is considered that people with a more positive attitude and a higher level of dental knowledge have better tooth brushing practice^60^. In the fsQCA positive solution, the higher coverage solution (C1 in Table 4) show that individual self-efficacy, oral knowledge and attitude can be considered important conditions affecting OHRQoL, but the results do not reflect the impact of practice. This is slightly different from previous studies^61^. The possible reason is that Chinese college students in this study are still in the sensitive period of constructing oral health practice, have strong plasticity, and are influenced by knowledge and attitude. Therefore, the results of this study support the core effect of the combination of knowledge, attitude, and self-efficacy on OHRQoL, which is consistent with previous results^62^. It is believed that patients with high quality of life have a high level of knowledge, a positive attitude and a greater sense of self-efficacy^63^.

Social support indirectly affects OHRQoL through the intermediary role of self-efficacy and a wide range of multi-dimensional factors such as knowledge, attitude and practice (Table 2), this is similar to previous results^54^. The indirect effect of social support is partially supported in the fsQCA solution (C3 in Table 4), which affects OHRQoL through a combination of self-efficacy and knowledge. This is consistent with results from patients with diabetes, suggesting that social support and knowledge are key prerequisites for patients’ self-care behavior^64^, and that having better social support and a high level of knowledge can better promote self-care. However, the fsQCA solution (C2 in Table 4) indicates that a combination of good attitude and strong self-efficacy can also support a high level of OHRQoL in the absence of social support, which to a certain extent emphasizes the impact of individual attitudes on oral health outcomes. This is consistent with Canter et al.^62^.

Consistent with Bandura's social cognitive theory, the results of this study further confirm that strategies to improve self-efficacy are beneficial to the improvement of health-related quality (HRQoL)^65^. However, this contrasts with the findings of Grolnick, W., who suggested that when parents over-intervene in balancing the health needs of adolescents, it may reduce their self-efficacy and lead to rebellious behavior^66^. Researcher can also give positive incentives to groups who correctly implement behaviors so that individuals can gain a higher sense of self-efficacy to improve oral health. This is also consistent with the fact that the external environment, human behavior, and individual cognitive processes jointly influence personal activities in Bandura's social cognitive theory^51^. Therefore, training for self-efficacy and its predictor factors will be the key to helping teenagers improve or maintain OHRQoL.

The rich and complex configuration of fsQCA results can help researchers improve oral health. The higher coverage solution (Table 4, score C1) shows that good self-efficacy, a high level of oral health knowledge and a positive attitude can improve OHRQoL. Therefore, it is necessary to regularly publicize oral health knowledge and improve cognitive levels among college students, while exploring internal motivation and favorable expectations of the group to guide college students to adopt positive attitudes to deal with various oral conditions. When teenagers have a successful experience and gain confidence, their self-efficacy improves, which further promotes the improvement or maintenance of their own OHRQoL, thus forming a positive feedback effect. This is consistent with previous measures aimed at self-management and improving the quality of life of patients with asthma^63^.

In addition, the development of extensive social support within the youth group will also help to improve the OHRQoL level. Researchers can obtain more extensive social support by encouraging subjects to increase social connections and communication activities, or through specific interventions to enhance subjects' perception of social support, to achieve higher self-efficacy and improve their quality of life. This is consistent with interventions to enhance perceived social support (PSS) and self-efficacy of patients with periodontitis, thereby improving their HRQoL after treatment^23^. At the same time, the OHRQoL of adolescents can be influenced from the social level by changing the public's concept of oral health, which is consistent with the strategy for the prevention of chronic diseases^67^.

In summary, the results obtained from the comprehensive application of PLS-SEM and fsQCA not only enrich the theoretical and practical value of the complex causal relationship between OHRQoL and multiple factors, but also lay a foundation for related research on the influencing factors of OHRQoL and provide a practical basis for the development of interventions to improve OHRQoL. However, this study also has some limitations. The study sample comprises college students aged 17–23, which may limit the generalizability of the conclusions. Furthermore, Lisa M. Jamieson's study noted that the cross-sectional nature of the findings does not establish causality^68^. To fully address this limitation, a longitudinal study design is necessary to investigate the influence of confounding factors on self-efficacy and oral health-related quality of life. Such a study would help establish a causal relationship between these two factors.

## Conclusions

Combining the advantages of PLS-SEM and fsQCA, this paper analyzed factors related to social support, oral health knowledge, attitude, practice, and self-efficacy that affect the oral health of college students. The results show that self-efficacy is the core factor affecting OHRQoL, and has an intermediary effect between other factors and OHRQoL. Social support acts widely on other influencing factors and has a positive effect on OHRQoL. While partially verifying the results of SEM, fsQCA suggests that other influencing factors are related to the complex configuration of OHRQoL. Among them, the combination of self-efficacy, oral knowledge, and attitude has greater coverage and a greater impact on OHRQoL. Therefore, the key to improving an individual college student’s OHRQoL is to improve self-efficacy. Under this premise, regular education to improve oral health understanding and guide them to actively deal with their own oral conditions will help to improve or maintain good OHRQoL. At the same time, developing extensive social support from around the college students group will also help to improve OHRQoL at a group level.

## Supplementary Information

Supplementary Information.

## Data Availability

The datasets used and/or analysed during the current study are available from the corresponding author on reasonable request.
