# Supplementary material for: Social support, oral health knowledge, attitudes, practice, self-efficacy and oral health-related quality of life in Chinese college students
Source: Sci Rep. 2023 Jul 29;13:12320. doi: 10.1038/s41598-023-39658-6 (PMC10387080; doi:10.1038/s41598-023-39658-6)
Supplement: Supplementary file 1 — Supplementary Information. [file 41598_2023_39658_MOESM1_ESM.pdf]

Table 1 The Constructed Items Measurement Model. (See end of article)

Constructs/items	Outer loading
**Social support (CR = 0.876, CA = 0.832, AVE = 0.543)**	
There is a special person with whom I can share my joys and sorrows (SS1)	0.718
I have a special person who is a real source of comfort to me (SS2)	0.708
I get the emotional help and support I need from my family (SS3)	0.708
My family is willing to help me make decisions (SS4)	0.688
I can count on my friends when things go wrong (SS5)	0.789
I can talk about my problems with my friends (SS6)	0.803
**Self-efficacy (CR = 0.921, CA = 0.905, AVE = 0.462)**	
I go to the dentist for treatment of periodontal disease (SE1)	0.774
I cooperate with my dentist and hygienist for treatment of periodontal disease (SE2)	0.824
I visit my dentist regularly, even after treatment is completed, to prevent recurrence (SE3)	0.785
I have regular checkups even when I am busy with work or housework (SE4)	0.750
I have regular checkups even when my mind is not relaxed (SE5)	0.785
I brush my teeth as instructed (SE6)	0.599
I brush my teeth carefully and thoroughly (SE7)	0.623
I brush the border between the teeth and gums (SE8)	0.671
I move the toothbrush with a short, quick motion (SE9)	0.736
I take time to brush my teeth carefully (SE10)	0.740
I try not to spend too much time eating (SE11)	0.323
I eat my meals at fixed times during the day (SE12)	0.563
I try to eat a well-balanced diet (SE13)	0.641
I try not to eat too many sweets (SE14)	0.524
**Knowledge (CR = 0.944, CA = 0.929, AVE = 0.739)**	
Plaque can cause tooth decay and periodontal disease, including gingivitis and periodontitis (K1)	0.86
Fluoride toothpaste can prevent caries (K2)	0.847
A regular dental deep cleaning using the ultrasonic dental scaler can maintain good oral hygiene (K4)	0.878
In the early stages of gingivitis, the gums bleed when brushing or biting hard objects (K5)	0.9
Caries are often characterized by irritation, biting discomfort or pain (K7)	0.898
Halitosis is mainly caused by oral diseases (K8)	0.765
**Attitude (CR = 0.895, CA = 0.844, AVE = 0.681)**	
Do you think eating sweets (such as cakes, biscuits and juices) will not cause tooth decay? (A1)	0.797
Do you think eating carbonated drinks will not cause tooth decay? (A2)	0.757
Do you think regular cleaning is good for oral health? (A3)	0.869
Do you think oral diseases can be prevented? (A4)	0.873
**Practice (CR = 0.883，CA = 0.847，AVE = 0.485)**	
Do you brush your teeth more than twice a day? (P1)	0.606
Is your brushing time ≥ 3 min each time? (P2)	0.737
Do you often use fluoride toothpaste? (P3)	0.682
Do you think regular oral check-ups are good for oral health? (P4)	0.62
Do you change a toothbrush every three months? (P5)	0.731
Do you often use dental floss (or an interdental brush) to help clean your teeth? (P6)	0.683
Is your brushing method recommended by the Chinese association of stomatology as "horizontal vibrating brush method"? (P7)	0.772
Do you often gargle after meals? (P8)	0.726
**OHRQoL (CR = 0.976，CA = 0.873，AVE = 0.748)**	
Have you had trouble pronouncing any words because of problems with your teeth, mouth or dentures? (I1)	0.825
Have you felt that your sense of taste has worsened because of problems with your teeth, mouth or dentures? (I2)	0.854
Have you had painful aching in your mouth? (I3)	0.589
Have you found it uncomfortable to eat any foods because of problems with your teeth, mouth or dentures? (I4)	0.81
Have you been self-conscious because of your teeth, mouth or dentures? (I5)	0.826
Have you felt tense because of problems with your teeth, mouth or dentures? (I6)	0.876
Has your diet been unsatisfactory because of problems with your teeth, mouth or dentures? (I7)	0.894
Have you had to interrupt meals because of problems with your teeth, mouth or dentures? (I8)	0.896
Have you found it difficult to relax because of problems with your teeth, mouth or dentures? (I9)	0.918
Have you been a bit embarrassed because of problems with your teeth, mouth or dentures? (I10)	0.887
Have you been a bit irritable with other people because of problems with your teeth, mouth or dentures? (I11)	0.921
Have you had difficulty doing your usual jobs because of problems with your teeth, mouth or dentures? (I12)	0.934
Have you felt that life in general was less satisfying because of problems with your teeth, mouth or dentures? (I13)	0.92
Have you been totally unable to function because of problems with your teeth, mouth or dentures? (I14)	0.903

SS, social support; SE, social efficacy; K, knowledge; A, attitude; P, practice
